# Effect of Capsular Closure on Outcomes of Hip Arthroscopy for Femoracetabular Impingement: A Systematic Review and Meta‐analysis

**DOI:** 10.1111/os.12717

**Published:** 2020-06-10

**Authors:** Liang Liu, Yan Zhang, Qi Gui, Feng Zhao, Xue‐Zhen Shen, Xing‐Huo Zhang, Xiao‐Peng Cong, Ya‐Kui Zhang

**Affiliations:** ^1^ Department of Sports Medicine Beijing LUHE Hospital Capital Medical University Beijing China; ^2^ Department of Education Beijing LUHE Hospital Capital Medical University Beijing China; ^3^ Department of Orthopedic Center Beijing LUHE Hospital Capital Medical University Beijing China

**Keywords:** Capsular closure, FAI, Hip arthroscopy, Meta‐analysis, Outcomes

## Abstract

**Objective:**

To evaluate the effect of hip arthroscopy with or without capsular closure in femoracetabular impingement (FAI) by meta‐analysis.

**Methods:**

Pertinent studies were identified by searching Pubmed, EMBASE databases with the last search update on 16 February 2020. Studies that reported hip arthroscopy for FAI were collected. Meta‐analysis was performed by the use of Review Manager 5.3 software. The odds ratios (OR) and mean differences (MD) were used to compare dichotomous and continuous variables. Additionally, the I^2^ was used to assess heterogeneity among studies, and the fixed‐effects model or the random‐effects model was selected for the quantitative analysis. Outcomes were evaluated by forest plots. For statistical analysis, *P* < 0.05 was considered significant.

**Results:**

There was no significant difference among the preoperative mHHS (MD = –2.66，95% CI [−7.25, 1.92], I^2^ = 80%, *P* = 0.25), preoperative (MD = ‐4.94, 95% CI [−11.56, 1.67], I^2^ = 50%, *P* = 0.14) and postoperative HOS‐SSS (MD = ‐1.00, 95% CI [−6.98, 4.98], I^2^ = 66%, *P* = 0.74), patient satisfaction (MD = 0.03, 95% CI [−0.25, 0.31], I^2^ = 19%, *P* = 0.84; OR = 0.94, 95% CI [0.59, 1.50], I^2^ = 0%, *P* = 0.78), complications (OR = 1.23, 95%CI [0.56, 2.67], I^2^ = 0%, *P* = 0.61), revisions (OR = 1.77, 95% CI [0.87, 3.60], I^2^ = 36%, *P* = 0.11), and surgery time (SMD = –0.38, 95% CI [−1.16, 0.40], I^2^ = 92%, *P* = 0.34) between the capsule closure group and the non‐closure group. For the comparison of postoperative mHHS (MD = –2.66, 95% CI [−7.25, 1.92], I^2^ = 80%, *P* = 0.25) and HOS‐ADL (MD = ‐4.20, 95% CI [−5.75, −2.65], I^2^ = 24%, *P* < 0.00001), the score of the non‐closure group was significantly better than that of the closure group.

**Conclusions:**

Remain capsule unclosed after hip arthroscopy for FAI may, to some extent, has a better postoperative functional score than the non‐closure treatment.

## Introduction

Due to the abnormal morphology and structure of the femur and acetabulum, repeated impacts of the proximal end of the femoral neck on the acetabular lip and its adjacent cartilage are important causes of adult hip pain and limited movement. This type of impact phenomenon is known as femoracetabular impingement (FAI)^1^.

The stability of the hip depends on the restraint of the capsule, the complex structure of bone and cartilage between the proximal femur and the acetabulum^2^. Among them, the joint capsule is composed of the iliofemoral, pubofemoral, ischiofemoral ligaments, zona orbicularis, and iliocapsularis, which is an important factor for the stability of the natural joint, guaranteeing the stability of the static and dynamic motion of the bone and joint around the hip joint^3^.

Treatment for FAI syndrome mainly includes non‐surgical and surgical options. The use of open suture capsulorrhaphy for hip instability has been reported for decades^4^, ^5^. In open FAI surgery, restoration of the physiological “sealing mechanism” of the acetabular labrum, as well as the normal hip morphology, has been a primary goal of hip surgeons^6^. Nowadays, arthroscopic surgery has become a preferred treatment option for the management of FAI. In contrast, arthroscopic FAI therapy has placed less emphasis on the restoration of hip capsule integrity. In the process of hip arthroscopy, the first step is to establish the anterolateral and mid‐anterior portals by using a safe access technique. In addition, the essential step in this process is the incision of the joint capsule. To get a better view under the arthroscope, most surgeons extended capsulotomies or even focal capsulectomies to achieve the same goals as open hip surgery^7^. However, several studies have suggested that routine capsular closure should be performed at the end of hip arthroscopy cases^8^, ^9^. As the usage rate of hip arthroscopy has increased considerably in recent years, increasing controversy has arisen about the defect of the unrepaired capsule. Although the joint capsule has an important physiological function, it is difficult to repair the joint capsule intraoperatively and the operation time is prolonged and the risk of complications is increased. In view of this, we conducted this meta‐analysis on existing relevant studies to compare the therapeutic effect between closing and not closing the joint capsule, therefore provide certain reference for clinical diagnosis and treatment.

## Materials and Methods

### *Search Strategy*

In accordance with the research aim, two authors searched relevant documents of PubMed and EMBASE with the last search update on 16 February 2020. The medical subject headings and keywords included: (((((capsule) OR capsules) OR capsular) OR capsular)) AND ((((((((((Femoroacetabular impingement) OR FAI) OR Impingement, Femoracetabular) OR Impingements, Femoracetabular) OR Impingements, Femoroacetabular) OR Impingement, Femoroacetabular) OR Impingement, Femoro‐Acetabular) OR Impingements, Femoro‐Acetabular)) AND ((((((Hips) OR Hip) OR Coxa) OR Coxas)) AND (((((Arthroscopies) OR Arthroscopic) OR Arthroscope) OR Arthroscopes) OR Arthroscopy))), and the keywords we used are combination of mesh terms and free terms for Pubmed and EMBASE. Read the full text of the articles which met the inclusion criteria carefully and extract relevant data for a comprehensive assessment. All included studies were determined on 20 February 2020.

### *Inclusion Criteria and Exclusion Criteria*

Inclusion criteria: (i) P (participants): FAI was definitely diagnosed, age and gender were not limited, post‐operation follow‐up for no less than 1 year; (ii) I (intervention): all the reported patients underwent hip arthroscopic surgery; (iii) C (comparison): the experimental subgroups reported in the article included capsular closure and non‐closure in arthroscopic surgery; (vi) O (outcome): at least one of the following outcomes was reported; (v) Study type: randomized controlled trials (RCTs) and non‐randomized controlled trials (nRCTs) on hip arthroscopy surgery of FAI with clear description of inclusion and outcome indicators, in Chinese or English.

Exclusion Criteria: (i) animal experiments or *in vitro* human cadaveric biomechanical studies; (ii) the original data or valid data cannot be obtained by contacting the author; (iii) duplicate reports, only abstracts, reviews, clinical practice guidelines, non‐comparative studies, and case reports.

### *Data Extraction*

Three authors (Liang Liu, Yan Zhang, and Qi Gui) independently screened the text, extracted eligible data, and reached conformity for all items. In case of disagreement, the fourth researcher (Ya‐Kui Zhang) assisted in solving the problem, and the lack of information was supplemented by contacting the author as much as possible. During the screening, title and abstract were read first, and the full text was read further to determine whether to include it or not after the obviously irrelevant literature was excluded. The information extracted from all primary research included author name, year of publication, titles, age, gender, study design, sample size, duration of follow‐up, and outcome parameters. The number of samples and positive cases of each study group were extracted for dichotomous data, and the number of samples, mean and standard deviation (SD) of each study group were extracted for continuous data.

### *Outcomes*

The following outcomes were extracted from the included studies: the modified Harris Hip Score (mHHS), Hip Outcome Score–Sport‐Specific Subscale (HOS‐SSS), Hip Outcome Score–Activities of Daily Living (HOS‐ADL), patient satisfaction, satisfaction rate, complications number, revisions number, and surgery time.

### *Modified Harris Hip Score*

mHHS is a multidimensional clinician‐reported outcome measure that contains seven items covering pain, function (gait), and functional activities^10^. The function includes limp, support, and distance, and the functional activities contain stairs, sock/shoes, sitting, and public transportation. It is considered as an ideal tool for the evaluation of patients who had undergone hip arthroscopy^11^.

### *Hip Outcome Score*

HOS is used to assess the outcome of treatment intervention for individuals with FAI, which includes two subscales: the activities of daily living (HOS‐ADL) and sport‐specific subscale (HOS‐SSS). The ADL subscale covers 19 items about basic daily activities, and the sports‐specific subscale includes nine items about higher level activities which required in athletics^12^. HOS‐ADL subscale focuses on a wide range of functions from small activities such as standing and sitting, to more demanding activities like twisting, pivoting, and squatting on the affected leg. HOS‐SSS looks at the ability of an individual to perform specific errands such as running or swinging items. This scale is about activities using their normal technique and includes movements like lateral, cutting motions, starting and stopping quickly.

### *Patient Satisfaction Scale*

Patient satisfaction scale is a numerical record of patient satisfaction with the outcome of surgery (0, not satisfied at all and 10, completely satisfied). When the patient satisfaction score >7, the patient was considered to be satisfied with the operation effect, otherwise, the patient was not satisfied. The percentage of satisfied patients in all patients included in the statistics is patient satisfaction rate. The number of postoperative complications in the two groups was counted, which includes numbness, skin rash, infection, lower limb deep‐vein thrombosis, heterotopic ossification, and nerve injury. Revision is the number of patients who needed secondary surgery after their first hip arthroscopy.

### *Quality Assessment*

We used Cochrane collaborative network quality assessment tool to evaluate the bias of the included RCT study, including: random sequence generation (selection bias); allocation concealment (selection bias); blinding of participants and personnel (performance bias); blinding of outcome assessment (detection bias); incomplete outcome data (attrition bias); selective reporting (reporting bias); other bias. For the non‐RCT study, the quality of the included study was also assessed by two independent authors using the methodological index for non‐randomized studies (MINORS). The scale has a total of 24 points, including: clearly stated aim; inclusion of consecutive patients; prospective collection of data; endpoints appropriate to the aim of the study; unbiased assessment of the study endpoint; follow‐up period appropriate to the aim of the study; loss to follow up less than 5%; prospective calculation of the study size; an adequate control group; contemporary groups; baseline equivalence of groups; and adequate statistical analyses.

### *Data Analysis*

The statistical analysis of the studies was performed with RevMan5.3 software. Odds ratio (OR) was used as an effective index for dichotomous data. Mean difference (MD) was used for continuous data, and 95% confidence intervals (CIs) were generated and assessed. A probability of *P* < 0.05 was considered to be statistically significant. I^2^ was utilized to evaluate the heterogeneity of the selected study. I^2^ > 50% represents high heterogeneity, and the random effect model was used for meta‐analysis. Otherwise, the fixed‐effect model was used for meta‐analysis. For less than 10 studies were assessed the possibility of publishing bias was not evaluated.

## Results

### *Study Selection and Characteristics*

**Fig 1:**
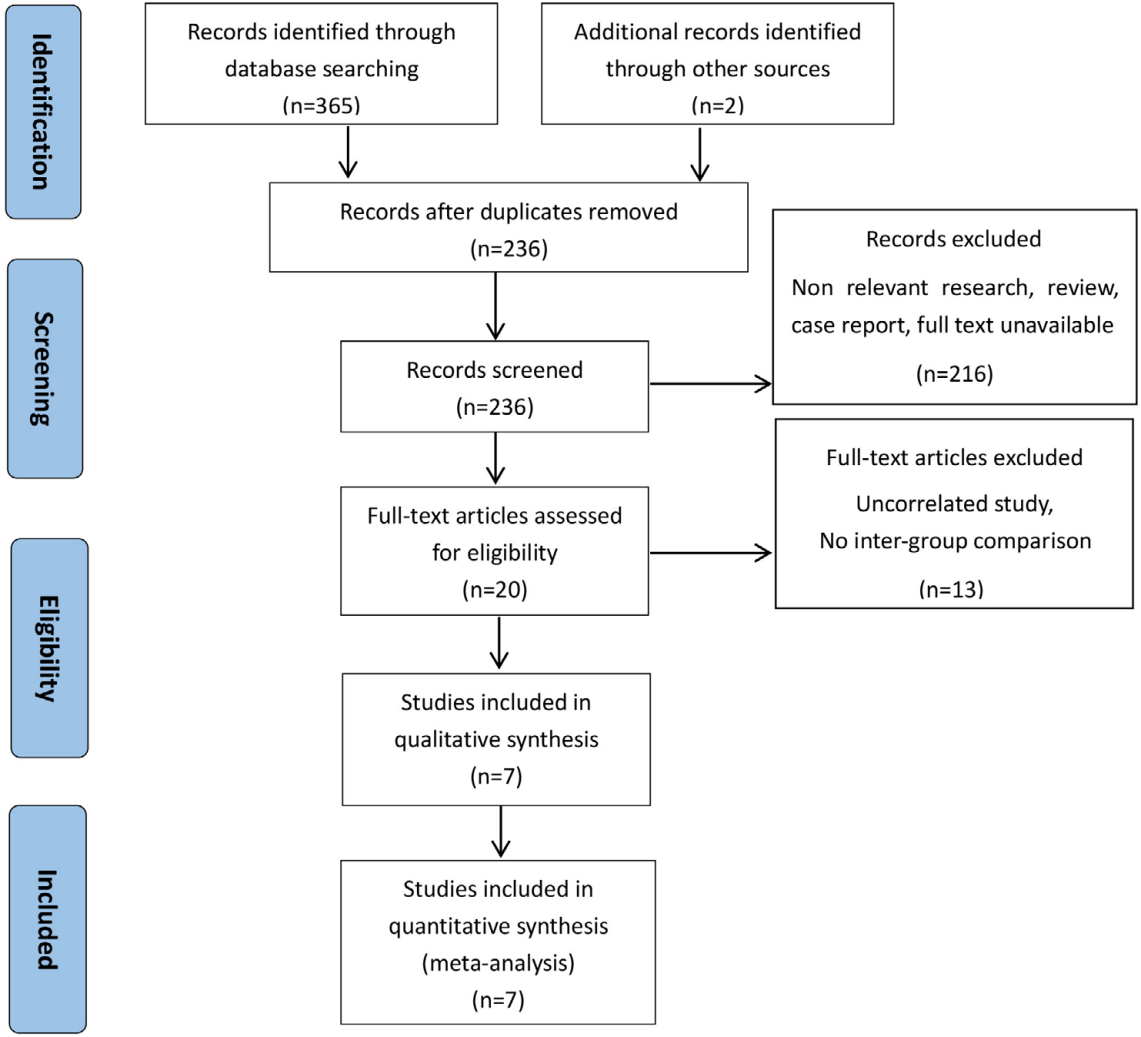
The flow diagram of the selection of eligible studies.

A preliminary total of 365 studies were identified from PubMed and EMBASE, due to the lack of available data, two high‐quality Chinese studies were included. Endnote 7.8 Software was used to screen out duplicate literature, 129 studies were excluded. After screening titles and abstracts, 216 studies were excluded, of which, 30 reviews or systematic review, 13 case reports, 11 articles cannot get the full text, and the remaining 20 articles were retrieved for full‐text review. Then, we excluded 13 articles based on inclusion criteria, which did not have relevant outcomes. Ultimately, seven studies^2^, ^3^, ^13^, ^14^, ^15^, ^16^, ^17^ were finally included in the meta‐analysis. Figure 1 shows the flow diagram of the included studies.

A total of 923 FAI patients after hip arthroscopy were included, of which 505 patients were treated with hip arthroscopy without capsular closure and 418 patients were in the capsular closure group (control group). The main characteristics of the studies identified are shown in Table 1.

**TABLE 1:** The main characteristics of studies included in the Meta‐analysis

Study	Year	Design	Sample, N			Age (years)		Sex (female)		BMI		Follow‐up（month）		Main outcomes	MINORS
			Total	Non‐closure	Capsular closure	Non‐closure	Capsular closure	Non‐closure	Capsular closure	Non‐closure	Capsular closure	Non‐closure	Capsular closure		
Amar *et al*.	2015	non‐RCT	100	50	50	36.7 ± 16.05	38.2 ± 13.86	19	20	NR	NR	12.1 ± 0.53	13.2 ± 0.65	Sugery time /revision	20
Atzmon *et al*.	2019	RCT	64	29	35	37.6 ± 15.24	38.1 ± 13.9	13	14	NR	NR	60.7	40.4	Satisfaction	
Domb *et al*.	2015	non‐RCT	403	235	168	42.31 ± 12.39	29.4 ± 12.36	97	136	26.75 ± 4.91	22.94 ± 3.84	26.76 ± 4.32	25.08 ± 3.12	mHHS/HOS‐ADL/	21
														HOS‐SSS/Satisfaction	
Domb *et al*.	2018	non‐RCT	130	65	65	37.7 ± 12.6	36.8 ± 12.4	47	47	24.4 ± 3.8	24.1 ± 3.3	75.7 ± 8.6	64.8 ± 4.2	mHHS/HOS‐SSS/Satisfaction/	19
														complication/revision	
Frank *et al*.	2014	RCT	64	32	32	32.87 ± 9.84	32.65 ± 10.16	20	20	24.98 ± 3.19	24.75 ± 3.77	30.1 ± 2.9	29.7 ± 2.5	mHHS/HOS‐ADL/HOS‐SSS/	
														Satisfaction/Sugery time	
Pan *et al*.	2019	RCT	60	30	30	39.57 ± 10.49	37.2 ± 10.72	16	15	NR	NR	NR	NR	mHHS/Satisfaction/sugery time	20
Chen *et al*.	2019	non‐RCT	102	64	38	38.59 ± 11.27	37.64 ± 11.10	36	21	21.95 ± 2.66	21.28 ± 2.52	NR	NR	mHHS/HOS‐ADL/complication/	
														Sugery time	

RCT, randomized controlled trials; N, number; BMI, body mass index; NR, not reported data; MINORS, methodological index for non‐randomized studies; mHHS, Modified Harris Hip Score; HOS‐ADL, Hip Outcome Score–Activities of Daily Living; HOS‐SSS, Hip Outcome Score–Sport‐Specific Subscale

### *Quality Assessment*

**Fig 2:**
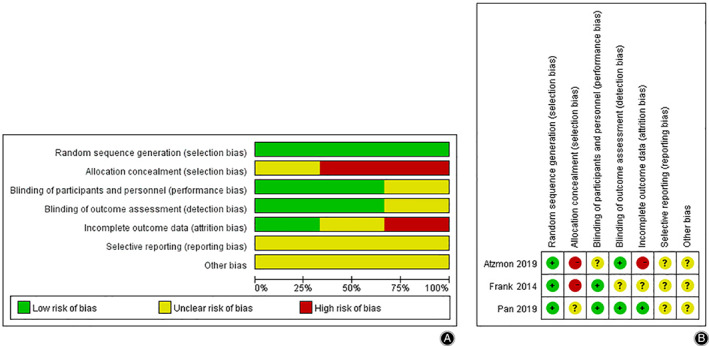
(A) Risk of bias graph: review authors' judgments about each risk of bias item presented as percentages across all included studies. (B) Risk of bias summary: review authors' judgments abou teach risk of bias item for each included study.

Among the included studies, there were four non‐RCT and three RCT. For RCTs we used the Cochrane collaborative network quality assessment tool, and for non‐RCTs we used MINORS to evaluate the quality. The bias of RCTS mainly exists in selective reporting and other bias. Four non‐RCT studies were conducted using the MINORS evaluation criteria, one paper scored 21 points, two papers scored 20 points, and one paper scored 19 points. The penalty points are in the prospective data collection and blind evaluation sections. Generally, more than 16 points were included in the study, and the articles selected in this meta‐analysis all met the requirements. The inclusion of the quality evaluation of the study is shown in Table 1 and Fig. 2.

### *Outcomes of Meta‐analysis*

#### *Modified Harris Hip Score (mHHS)*

**Fig 3:**
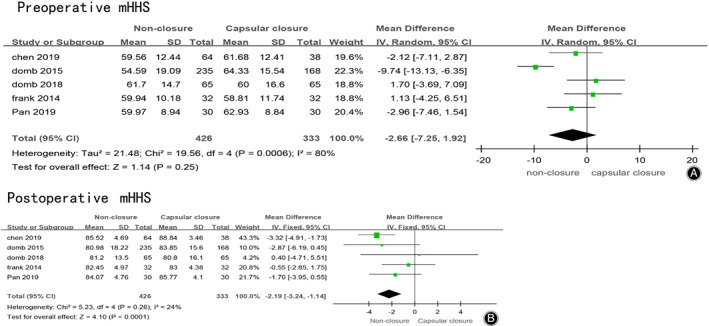
(A) Forest plots of preoperative mHHS in non‐closure and capsular closure group in hip arthroscopy for FAI. (B) Forest plots of postoperative mHHS in non‐closure and capsular closure group in hip arthroscopy for FAI.

Five studies reported the scores of mHHS before hip arthroscopy in the non‐closure group and the capsular closure group, 759 patients were included. The meta‐analysis results were shown in Fig. 3A, which showed that there was no significant difference in preoperative mHHS between the two groups (MD = –2.66，95% CI [−7.25, 1.92], I^2^ = 80%, *P* = 0.25).

Five studies reported the scores of mHHS after hip arthroscopy in the non‐closure group and the capsular closure group, 759 patients were included. The meta‐analysis results were shown in Fig. 3B, which showed a significant difference in postoperative mHHS between the two groups. Postoperative MHHS score of the non‐closed group was better than that of the capsular closed group (MD = −2.19，95% CI [−3.24, −1.14], I^2^ = 24%, *P* < 0.0001).

#### *Hip Outcome Score–Activities of Daily Living (HOS‐ADL)*

**Fig 4:**
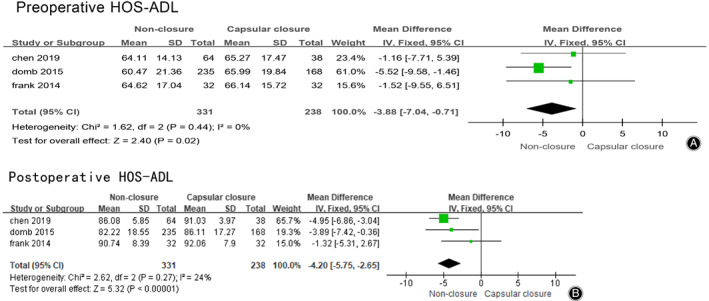
(A) Forest plots of preoperative HOS‐ADL in non‐closure and capsular closure group in hip arthroscopy for FAI. (B) Forest plots of postoperative HOS‐ADL in non‐closure and capsular closure group in hip arthroscopy for FAI.

Three studies reported the scores of HOS‐ADL before hip arthroscopy in the non‐closure group and the capsular closure group, and a total of 569 patients were included. The meta‐analysis results were shown in Fig. 4A. Preoperative HOS‐ADL score of the non‐closed group was better than that of the closed group (MD = –3.88，95% CI [−7.04, −0.71], I^2^ = 0%, *P* = 0.02).

Three studies reported the scores of HOS‐ADL before hip arthroscopy in the non‐closure group and the capsular closure group, 569 patients were included. The meta‐analysis results were shown in Fig. 4B, which showed no significant difference in postoperative HOS‐ADL between the two groups. Postoperative HOS‐ADL score of the non‐closed group was better than that of the closed group (MD = –4.20, 95% CI [−5.75, −2.65], I^2^ = 24%, *P* < 0.00001).

#### *Hip Outcome Score–Sport‐Specific Subscale (HOS‐SSS)*

**Fig 5:**
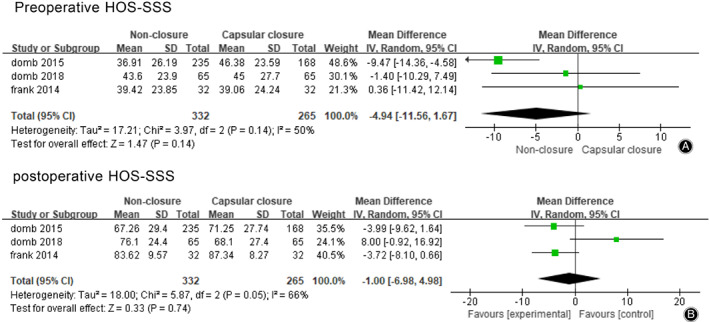
(A) Forest plots of preoperative HOS‐SSS in non‐closure and capsular closure group in hip arthroscopy for FAI. (B) Forest plots of postoperative HOS‐SSS in non‐closure and capsular closure group in hip arthroscopy for FAI.

Three studies reported the scores of HOS‐SSS before hip arthroscopy in the non‐closure group and the capsular closure group, 597 patients were included. The meta‐analysis results were shown in Fig. 5A. Meta‐analysis showed no significant difference in preoperative HOS‐SSS between the two groups (MD = –4.94，95% CI [−11.56, 1.67], I^2^ = 50%, *P* = 0.14).

Three studies reported the scores of HOS‐SSS before hip arthroscopy in the non‐closure group and the capsular closure group, 597 patients were included. The meta‐analysis results were shown in Fig. 5B, which showed that there was no significant difference in postoperative HOS‐SSS between the two groups (MD = –1.00，95% CI [−6.98, 4.98], I^2^ = 66%, *P* = 0.74).

### *Revision and Complication*

**Fig 6:**
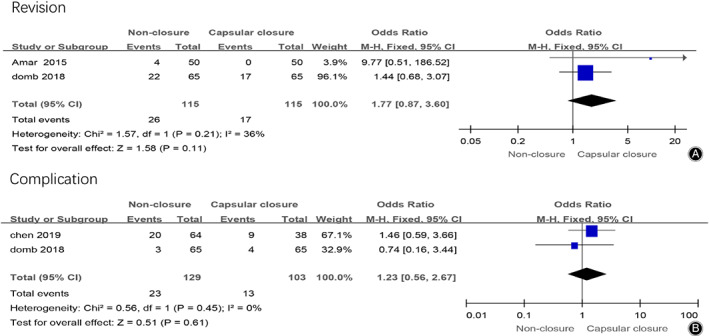
(A) Forest plots of postoperative revision in non‐closure and capsular closure group in hip arthroscopy for FAI. (B) Forest plots of postoperative complications in non‐closure and capsular closure group in hip arthroscopy for FAI.

Two studies reported the patient second operation rate after hip arthroscopy in the non‐closure group and the capsular closure group, 230 patients were included. The results of meta‐analysis were shown in Fig. 6A, which showed that there was no significant difference in postoperative patient revision between these two groups (OR = 1.77, 95% CI [0.87, 3.60], I^2^ = 36%, *P* = 0.11).

Two studies reported the patient complication rate after hip arthroscopy in the non‐closure group and the capsular closure group, 232 patients were included. The meta‐analysis results were shown in Fig. 6B, which showed that there was no significant difference in postoperative patient satisfaction rate between the two groups (OR = 1.23, 95% CI [0.56, 2.67], I^2^ = 0%, *P* = 0.61).

### *Surgery Time*

**Fig 7:**
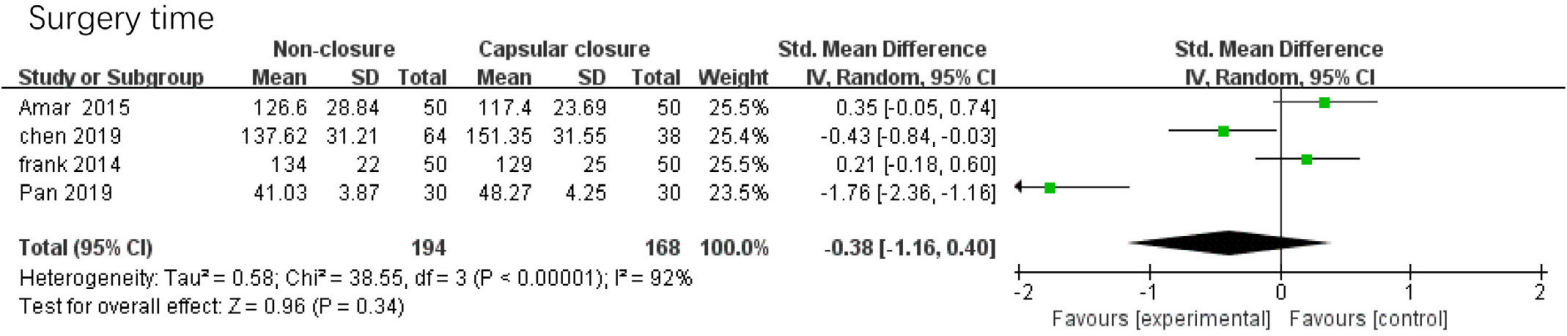
Forest plots of surgery time in non‐closure and capsular closure group in hip arthroscopy for FAI.

Four studies reported the patient satisfaction scale after hip arthroscopy in the non‐closure group and the capsular closure group, 362 patients were included. The meta‐analysis results were shown in Fig. 7, which showed that there was no significant difference in postoperative patient satisfaction between the two groups (SMD = –0.38, 95% CI [−1.16, 0.40], I^2^ = 92%, *P* = 0.34).

### *Patient Satisfaction Scale*

**Fig 8:**
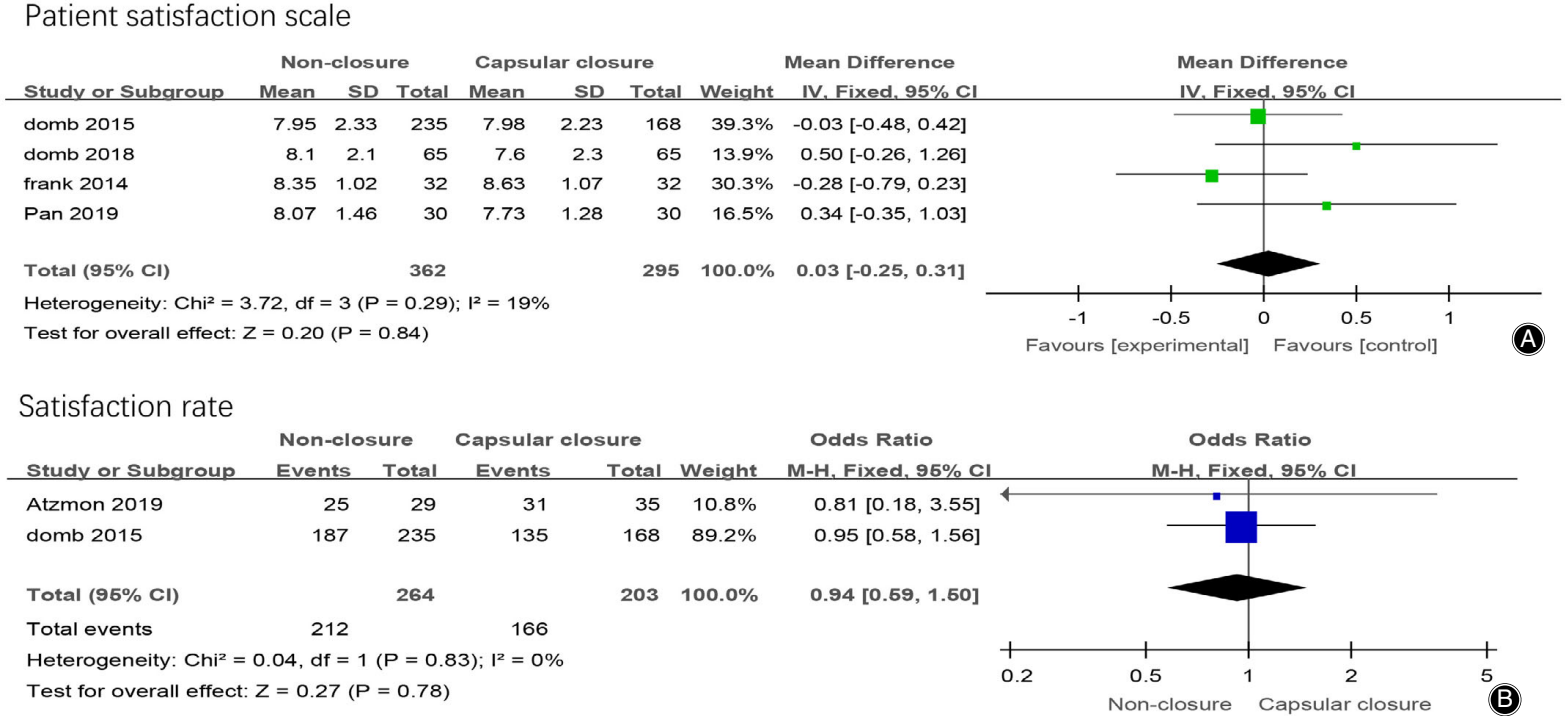
(A) Forest plots of postoperative patient satisfaction scale in non‐closure and capsular closure group in hip arthroscopy for FAI. (B) Forest plots of postoperative patient satisfaction rate in non‐closure and capsular closure group in hip arthroscopy for FAI.

Four studies reported the patient satisfaction scale after hip arthroscopy in the non‐closure group and the capsular closure group, 657 patients were included. The meta‐analysis results were shown in Fig. 8A, which showed that there was no significant difference in postoperative patient satisfaction between the two groups (MD = 0.03，95% CI [−0.25, 0.31], I^2^ = 19%, *P* = 0.84).

Two studies reported the patient satisfaction rate after hip arthroscopy in the non‐closure group and the capsular closure group, 467 patients were included. The meta‐analysis results were shown in Fig. 8B, which showed that there was no significant difference in postoperative patient satisfaction rate between the two groups (OR = 0.94, 95% CI [0.59, 1.50], I^2^ = 0%, *P* = 0.78).

### *Sensitivity Analysis*

#### *Modified Harris Hip Score (mHHS)*

After each study was eliminated one by one, the meta‐analysis of the combined effect size was performed again, and the results of the new combined effect size were compared with the results before the elimination. The preoperative mHHS results showed no significant difference, indicating low sensitivity and stable reliability (Table 2).

**TABLE 2:** Sensitivity analysis of preoperative mHHS

Excluded literature	MD	95%CI	I^2^ (%)	*P*‐value
no	−2.66	[−7.25, 1.92]	80	0.25
Pan 2019	−2.5	[−8.46, 3.46]	84	0.41
Frank 2014	−3.54	[−8.64, 1.56]	81	0.17
Domb 2018	−3.69	[−8.63, 1.26]	79	0.14
Domb 2015	−0.85	[−3.36, 1.67]	0	0.51
Chen 2019	−2.73	[−8.46, 3.01]	84	0.35

After each study was eliminated one by one, the meta‐analysis of the combined effect size was performed again, and the results of the new combined effect size were compared with the results before the elimination. Among them, heterogeneity of postoperative mHHS changed after the elimination of one item (Chen 2019), which is the reason for the large heterogeneity of this study. The source of heterogeneity may be the number of samples (Table 3).

**TABLE 3:** Sensitivity analysis of postoperative mHHS

Excluded literature	MD	95%CI	I^2^ (%)	*P*‐value
no	−2.19	[−3.24, −1.14]	24	<0.0001
Chen 2019	−1.33	[−2.72, 0.06]	0	0.06
Domb 2015	−2.12	[−3.22, −1.01]	41	0.002
Domb 2018	−0.3	[−3.37, −1.23]	29	<0.0001
Frank 2014	−2.62	[−3.80, −1.45]	0	<0.0001
Pan 2019	−2.33	[−3.51, −1.14]	40	0.0001

#### *Hip Outcome Score–Activities of Daily Living (HOS‐ADL)*

After each study was eliminated one by one, the meta‐analysis of the combined effect size was performed again, and the results of the new combined effect size were compared with the results before the elimination. Among them, heterogeneity of preoperative HOS‐ADL changed after the elimination of one item (Domb 2015), which is the reason for the large heterogeneity of this study. The source of heterogeneity may be the different outcome indicators (Table 4).

**TABLE 4:** Sensitivity analysis of preoperative HOS‐ADL

Excluded literature	MD	95%CI	I^2^ (%)	*P*‐value
no	−3.88	[−7.04, −0.71]	0	0.02
Chen 2019	−4.71	[−8.33, −1.09]	0	0.01
Domb 2015	−1.3	[−6.38, 3.77]	0	0.61
Frank 2014	−4.31	[−7.76, −0.86]	19	0.01

After each study was eliminated one by one, the meta‐analysis of the combined effect size was performed again, and the results of the new combined effect size were compared with the results before the elimination. The postoperative HOS‐ADL results showed no significant difference, indicating low sensitivity and stable reliability (Table 5).

**TABLE 5:** Sensitivity analysis of postoperative HOS‐ADL

Excluded literature	MD	95%CI	I^2^ (%)	*P*‐value
no	−0.32	[−0.49, −0.16]	79	0.0002
Chen 2019	−0.21	[−0.39, −0.02]	0	0.03
Domb 2015	−3.58	[−7.03, −0.13]	61	0.04
Frank 2014	−4.71	[−6.39, −3.03]	0	<0.00001

#### *Hip Outcome Score–Sport‐Specific Subscale (HOS‐SSS)*

After each study was eliminated one by one, the meta‐analysis of the combined effect size was performed again, and the results of the new combined effect size were compared with the results before the elimination. The preoperative HOS‐SSS results showed no significant difference, indicating low sensitivity and stable reliability (Table 6).

**TABLE 6:** Sensitivity analysis ofpreoperative HOS‐SSS

Excluded literature	MD	95%CI	I^2^ (%)	*P*‐value
no	−4.94	[−11.56, 1.67]	50	0.14
Domb 2015	−0.76	[−7.86, 6.34]	0	0.83
Domb 2018	−6.08	[−15.24, 3.08]	56	0.19
Frank 2014	−6.32	[−14.04, 1.39]	59	0.11

After each study was eliminated one by one, the meta‐analysis of the combined effect size was performed again, and the results of the new combined effect size were compared with the results before the elimination. Among them, heterogeneity of postoperative HOS‐SSS changed after the elimination of one item (Domb 2018), which is the reason for the large heterogeneity of this study. The source of heterogeneity may be the number of samples (Table 7).

**TABLE 7:** Sensitivity analysis of postoperative HOS‐SSS

Excluded literature	MD	95%CI	I^2^ (%)	*P*‐value
no	−1	[−6.98, 4.98]	66	0.74
Domb 2015	1.47	[−9.94, 12.88]	81	0.8
Domb 2018	−3.82	[−7.28, −0.36]	0	0.03
Frank 2014	1.49	[−10.22, 13.19]	80	0.8

#### *Surgery Time*

After each study was eliminated one by one, the meta‐analysis of the combined effect size was performed again, and the results of the new combined effect size were compared with the results before the elimination. The results of surgery time showed no significant difference, indicating low sensitivity and stable reliability (Table 8).

**TABLE 8:** Sensitivity analysis of surgery time

Excluded literature	SMD	95%CI	I^2^ (%)	*P*‐value
no	−0.38	[−1.16, 0.40]	92	0.34
Amar 2015	−0.64	[−1.64, 0.36]	93	0.21
Chen 2019	−0.38	[−1.49, 0.74]	95	0.51
Frank 2014	−0.59	[−1.67, 0.48]	94	0.28
Pan 2019	0.04	[−0.42, 0.51]	76	0.86

#### *Patient Satisfaction*

After each study was eliminated one by one, the meta‐analysis of the combined effect size was performed again, and the results of the new combined effect size were compared with the results before the elimination. The results of postoperative patient satisfaction showed no significant difference, indicating low sensitivity and stable reliability (Table 9).

**TABLE 9:** Sensitivity analysis of patient satisfaction

Excluded literature	MD	95%CI	I^2^ (%)	*P*‐value
no	0.03	[−0.12, 0.19]	14	0.67
Domb 2015	0.11	[−0.14, 0.35]	32	0.4
Domb 2018	−0.01	[−0.19, 0.16]	0	0.87
Frank 2014	0.07	[−0.10, 0.23]	0	0.42
Pan 2019	0.01	[−0.15, 0.17]	27	0.88

## Discussion

With the deepening understanding of the pathogenesis of FAI and the rapid development of arthroscopy technology, the application of hip arthroscopy in the treatment of FAI is becoming increasingly common, accompanied by the surgical methods and procedures becoming increasingly mature^18^. Presently, minimally invasive hip arthroscopy has become the standard operation option for FAI. Repairing the capsule requires operating among the strong muscles around the hip joint, prolongs surgery time, and increases the risk of complications, which make it difficult. Given this, the influence of this procedure on hip stability and the need to close the joint capsule during the operation turned into the focus of research in recent years.

In this study, a total of 923 patients were included in seven studies. Meta‐analysis was conducted to compare the clinical efficacy of hip arthroscopy in the treatment of FAI with or without closing the capsule. All the literature adopted in this study can proves that the mHHS score, HOS‐ADL score, and HOS‐SSS score were significantly improved in postoperative follow‐up after hip arthroscopy for FAI, regardless of whether the joint capsule was closed or not, indicating that the effect of hip arthroscopy for FAI was definite.

This study conducted a meta‐analysis on the postoperative score of hip arthroscopy by means of meta‐analysis and found that there was no statistical difference in the preoperative mHHS, preoperative and postoperative HOS‐SSS, postoperative satisfaction, complications, revisions and surgery time between the closed capsule and the non‐closed capsule groups. In postoperative mHHS and HOS‐ADL, the scores of the non‐closed group were significantly better than that of the closed group. Although the HOS‐ADL score before surgery was also statistically significant in the non‐closure group, compared with the *P*‐value of HOS‐ADL after surgery, the difference of the postoperative group was more obvious. The available data suggest that not closing the capsule after hip arthroscopy may, to some extent, result in a better postoperative functional score than closing the capsule. However, due to the limited number of relevant studies involved in this paper and the lack of large samples and big data, the above conclusions are only applicable to the current research results.

FAI syndrome has three types of morphology: cam morphology, pincer morphology, and mixed morphology. In 2013, Sankar *et al*. further describe FAI definition as “five essential elements”: (i) abnormal morphology of the femur and/or acetabulum; (ii) abnormal contact between these two structures; (iii) vigorous supraphysiological motion; (iv) repetitive motion resulting in the continuous insult; and (v) the presence of soft‐tissue damage^19^. In the normal state, the bony structure of the hip joint, acetabular labrum, and the joint capsule provide static constraint to the joint throughout a variety of physiological motions. The hip capsule is composed of four parts: pubofemoral, iliofemoral, ischiofemoral ligaments, and zona orbicularis, and the ischiofemoral ligaments control internal rotation in extension and flexion, the pubofemoral ligament controls external rotation, the iliofemoral ligament controls external rotation in flexion and both external and internal rotation in extension^20^. Meanwhile, dynamic stabilizers such as the rectus femoris, iliopsoas, and abductor complex also contribute to the maintenance of proper joint force‐coupled compression and kinematics that enhance hip joint stability^21^.

Notably, several studies have shown that 35% of patients had instability after arthroscopy^22^. Biomechanical studies have shown that capsules play an important role in hip stability, closed capsule restores hip kinematics better than leaving it unrepaired^23^, ^24^. Frank *et al*. also demonstrated that the partial repair group had a higher revision rate and lower patient‐reported outcomes than the complete repair group^13^.

In addition, routine repairing of the capsular in all patients may result in a higher incidence of postoperative stiffness. Some authors believe that in cases of hip joint stiffness or poor capsular compliance, capsulectomy may be a viable treatment option for some patients^25^, ^26^. In the case of subtle capsular laxity or athletic individuals without preoperative stiffness, capsular closure may reduce postoperative microinstability and accelerate the process of recovery. Some skeptics believe that it is difficult to suture the capsule under hip arthroscopy, which may therefore increase the operative time, damage surrounding tissues, and increase the possibility of complications. However, the statistical results of this present study showed that there was no statistical difference in postoperative complications, revisions, and surgery time between the closed and non‐closed joint capsule groups. Self‐factors of patients may have a considerable influence on outcomes. In this case, according to the different characteristics of the patient and the operative time, the choice of closure or non‐closure of the joint capsule is selected to better guarantee the operation effects.

### *Limitations*

This study has some limitations, including: (i) this study only searched the Chinese and English databases, but failed to retrieve and include the documents published in other languages; (ii) the quality of the included literature is generally not high, and the risks of methodological quality assessment are mostly unclear, which may have a certain impact on the research results of this systematic review; and (iii) the number of articles included is limited, only seven, and the number of subjects is relatively small. In sensitivity analysis, some key data may get different results after deleting a certain literature. With the increase of research reports on this subject in the future, there will be more convincing results.

### *Conclusion*

In conclusion, the treatment of FAI by hip arthroscopy can improve the patient's symptoms whether the joint capsule is closed or not. By means of meta‐analysis, we revealed that there was no significant statistical difference in the preoperative mHHS, preoperative and postoperative HOS‐SSS, patient satisfaction, complications, revision rates, and surgery time between the closed capsule and the non‐closed capsule groups. While in postoperative mHHS and HOS‐ADL, the score of the non‐closed group was significantly better than the closed capsule group. Patient related factors can have a considerable influence on the outcomes. The present meta‐analysis suggests that keeping the capsule unclosed after hip arthroscopy may result in a better postoperative functional score than closing the capsule.
